# Berry Phenolic Compounds Increase Expression of Hepatocyte Nuclear Factor-1α (HNF-1α) in Caco-2 and Normal Colon Cells Due to High Affinities with Transcription and Dimerization Domains of HNF-1α

**DOI:** 10.1371/journal.pone.0138768

**Published:** 2015-09-28

**Authors:** Luis M. Real Hernandez, Junfeng Fan, Michelle H. Johnson, Elvira Gonzalez de Mejia

**Affiliations:** 1 Department of Food Science and Human Nutrition, University of Illinois at Urbana-Champaign, Urbana, Illinois, 61801, United States of America; 2 Department of Chemistry, University of Illinois at Urbana-Champaign, Urbana, Illinois, 61801, United States of America; 3 College of Bioscience and Biotechnology, Beijing Forestry University, Beijing, 100083, China; 4 Division of Nutritional Sciences, University of Illinois at Urbana-Champaign, Urbana, Illinois, 61801, United States of America; University of Sassari, ITALY

## Abstract

Hepatocyte nuclear factor-1α (HNF-1α) is found in the kidneys, spleen, thymus, testis, skin, and throughout the digestive organs. It has been found to promote the transcription of various proteins involved in the management of type II diabetes, including dipeptidyl peptidase-IV (DPP-IV). Phenolic compounds from berries and citrus fruits are known to inhibit DPP-IV, but have not been tested for their interactions with wild-type HNF-1α. By studying the interactions of compounds from berries and citrus fruits have with HNF-1α, pre-transcriptional mechanisms that inhibit the expression of proteins such as DPP-IV may be elucidated. In this study, the interactions of berry phenolic compounds and citrus flavonoids with the dimerization and transcriptional domains of HNF-1α were characterized using the molecular docking program AutoDock Vina. The anthocyanin delphinidin-3-*O*-arabinoside had the highest binding affinity for the dimerization domain as a homodimer (-7.2 kcal/mol) and transcription domain (-8.3 kcal/mol) of HNF-1α. Anthocyanins and anthocyanidins had relatively higher affinities than resveratrol and citrus flavonoids for both, the transcription domain and the dimerization domain as a homodimer. The flavonoid flavone had the highest affinity for a single unit of the dimerization domain (-6.5 kcal/mol). Nuclear expression of HNF-1α was measured in Caco-2 and human normal colon cells treated with blueberry and blackberry anthocyanin extracts. All extracts tested increased significantly (P < 0.05) the nuclear expression of HNF-1α in Caco-2 cells by 85.2 to 260% compared to a control. The extracts tested increased significantly (P < 0.02) the nuclear expression of HNF-1α in normal colon cells by 48.6 to 243%. It was confirmed that delphinidin-3-*O*-glucoside increased by 3-fold nuclear HNF-1α expression in Caco-2 cells (P < 0.05). Anthocyanins significantly increased nuclear HNF-1α expression, suggesting that these compounds might regulate the genes HNF-1α promotes.

## Introduction

Hepatocyte nuclear factor-1α (HNF-1α) is a transcription factor that was originally thought to only be expressed in the liver, but has now been found to be expressed in the kidneys, pancreas, intestines, stomach, spleen, thymus, testis, and keratinocytes and melanocytes in human skin. It is composed of a dimerization domain (residues 1–32), a transcription domain (residues 203–276), and a transactivation domain (residues 281–631) [1]. The dimerization domain is involved in the dimerization of HNF-1α proteins, which is critical for DNA binding, and the dimerization domain is also the site of interaction with co-activators of HNF-1α [2]. The dimerization coactivator of HNF-1α (DCoH), for example, binds to the dimerization domain and stimulates HNF-1α activity [3]. The transcription domain binds to DNA, while the actions of the transactivation domain are still not well understood. To this date, only the dimerization domain and transcription domain of HNF-1α are well characterized, and have known crystallographic structures deposited in The RCBS Protein Data Bank (PDB, http://www.rcsb.org). Autosomal dominant mutations in HNF-1α are the cause of maturity onset diabetes of the young 3 (MODY3), which is the most common form of MODY [4]. Mutant HNF-1α constructs have been shown to have dominant-negative effects on the promotion of genes [5], indicating that mutations to HNF-1α can decrease expression of the genes it promotes and cause disease. HNF-1α has been recently shown to be involved in GLUT1 and GLUT2 transporter expression in pancreatic β-cells [6] and angiotensin-converting enzyme 2 gene expression in pancreatic islets [7].

HNF-1α is involved in various metabolic pathways of the digestive organs, such as being a transcriptional regulator of bile acid transporters in the intestine and kidneys [8]. Therefore, loss of HNF-1α functionality can cause metabolic disorders such as hypercholesterolemia. HNF-1α is also involved in the promotion of hepatic organic cation transporters which uptake certain classes of pharmaceuticals [9], hence loss of HNF-1α functionality in the liver can lead to drug metabolism problems. Aside from metabolic disorders, HNF-1α also regulates the expression of acute phase proteins, such as fibrinogen and interleukin 1 receptor, which are involved with inflammation [10]. One of the acute phase proteins HNF-1α regulates is C-reactive protein (CRP), which is used to monitor inflammation and possible progression of coronary heart disease.

HNF-1α has also been shown to promote the transcription of several proteins involved in the management of type II diabetes including dipeptidyl peptidase-IV (DPP-IV/CD26) [5, 11]. Type II diabetes is characterized by the development of insulin resistance and it is estimated to affect 90–95% of the United States diabetic population [12]. Current efforts to manage type II diabetes include lifestyle changes [12] and administration of pharmaceuticals including DPP-IV inhibitors [13]. DPP-IV is a serine protease that cleaves N-terminal amino acids in polypeptides such as glucagon-like peptide 1 (GLP-1), which stimulates insulin release [5]. By inhibiting DPP-IV, polypeptides such as GLP-1 are able to help lower blood glucose levels in patients with type II diabetes. Mechanisms of pre-transcriptional and transcriptional regulation of DPP-IV expression are still not well understood; therefore, studying the interactions of different compounds with HNF-1α would begin to elucidate pre-transcriptional mechanisms of DPP-IV regulation.

The stilbenoid resveratrol has been shown to closely interact with a mutated form HNF-1α (PDB: 2GYP) using computational docking methods, and is said to be a possible regulator of HNF-1α activity [14]. Resveratrol, along with other berry and citrus flavonoids, has also been shown to inhibit DPP-IV using both *in vitro* and *in silico* methods [15]. The effect of flavonoids from berries and citrus fruits on HNF-1α has not been studied, though several of these compounds have shown to have potential for positive health benefits [16–18]. In this study, the interactions of various berry phenolic compounds and citrus flavonoids with wild-type HNF-1α were analyzed computationally using the molecular docking program AutoDock Vina in order to gain insight on how these compounds might regulate HNF-1α. Wild-type HNF-1α was studied to assess the interactions of phenolic compounds with a form of HNF-1α commonly present in non-diabetic individuals and non-MODY3 diabetics. We hypothesized that the compounds tested, due to their small molecular size, would have a high affinity for HNF-1α allowing them to make strong interactions with HNF-1α. The expression of HNF-1α was also analyzed in Caco-2 and human normal colon cells treated with anthocyanin extracts after anthocyanins had a high affinity for HNF-1α. The anthocyanin extracts were mixtures of different anthocyanins as this is how they are usually consumed from fruits and vegetables. The anthocyanin extracts tested significantly increased expression of HNF-1α, and several of the studied compounds had high affinities with the dimerization and transcription domains of HNF-1α.

## Materials and Methods

### Molecular Docking

#### Ligand preparation

**Fig 1:**
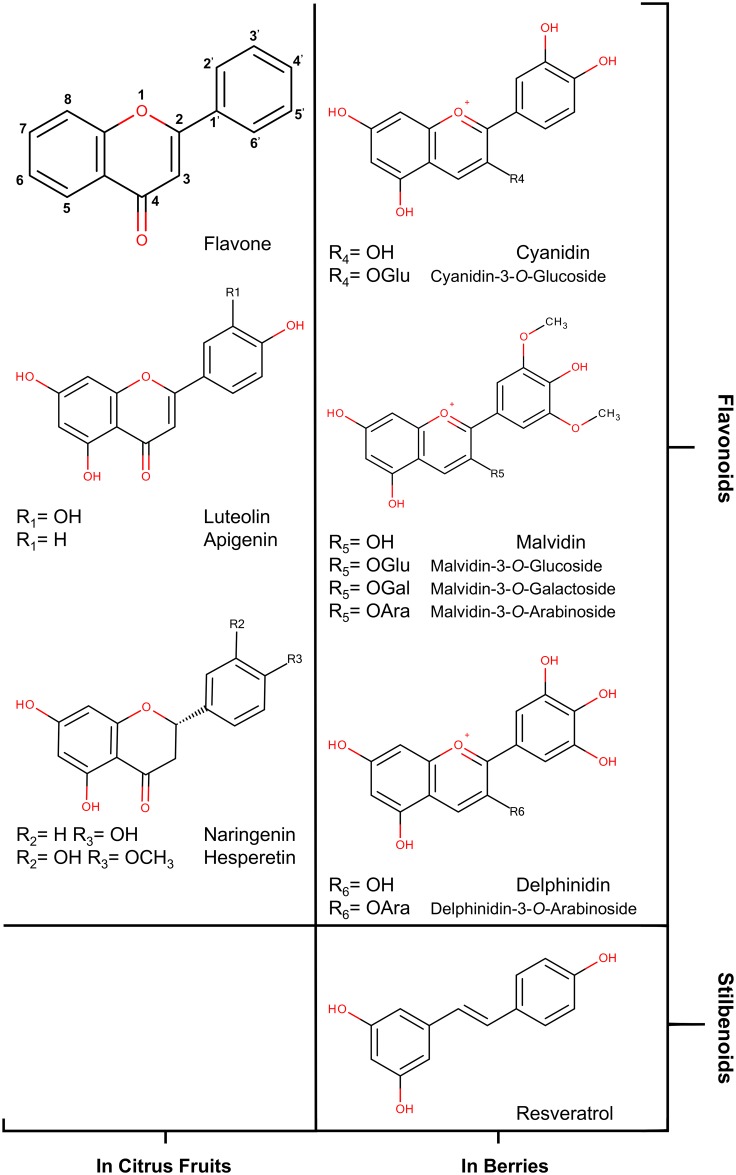
Molecular structures of berry and citrus compounds studied. Columns are divided by the source of each compound. The corresponding class of each compound is identified in the right margin. Numbering of atoms in the basic flavonoid structure is shown in the structure of flavone. Glycoside substituents are named by the first three letters of their name when defining R groups and spelled out in each compound’s name.

The structures of berry phenolic compounds and citrus flavonoids studied are shown in Fig 1. The stilbenoid resveratrol and the citrus flavonoids luteolin, apigenin, flavone, naringenin, and hesperetin were selected due to their low DPP-IV IC_50_ (μM) values compared to diprotin A, a known DPP-IV inhibitor [15]. Compounds in the flavonoid class of anthocyanins (cyanidin-3-*O*-glucoside, delphinidin-3-*O*-arabinoside, malvidin-3-*O*-arabinoside, malvidin-3-*O*-galactoside, and malvidin-3-*O*-glucoside) were selected because they were present in large concentrations (≥ 200mg cyanidin-3-*O*-glucoside equivalents/L) in blueberry and blackberry fermented anthocyanin extracts [19]. The non-glycosylated forms of anthocyanins, anthocyanidins (cyanidin, delphinidin, malvidin), were also evaluated to investigate the differences between anthocyanins and anthocyanidins.

A molecular file of each compound was obtained from Chemical Book (www.chemicalbook.com), except for delphinidin-3-*O*-arabinoside and malvidin-3-*O*-arabinoside which were drawn using Marvin Sketch (Marvin 5.10.0, 2012, ChemAxon, http://www.chemaxon.com). Molecular files were opened in Accelrys Discovery Studio 4.0 Visualizer (DS 4.0; Accelrys Software Inc., Discovery Studio Modeling Environment, Release 4.0, San Diego: Accelrys Software Inc., 2013) and had all heteroatoms removed. Ligands were added hydrogen atoms and applied the CHARMM22 force field. The DREIDING force field was then applied to all the ligands through the DreidingMinimize function in order to further minimize their intramolecular energy. Ligands were opened in AutoDockTools (version 1.5.6) [20] and were added Gasteiger charges. All rotatable bonds detected by AutoDockTools were kept rotatable for each ligand except for bonds that would break conjugation between π-orbitals such as between carbons 2 and 1’ when a π-bond was present between carbons 2 and 3 in a flavonoid (See atom numbering in Fig 1).

#### Hepatocyte nuclear factor- 1α structure preparation

The RCBS Protein Data Bank (http://www.rcsb.org/pdb) files for the wild-type transcription domain (PDB: 1IC8) and dimerization domain (PDB: 1G39) of hepatocyte nuclear factor-1α (HNF-1α) were opened in DS 4.0. Water molecules in each file were deleted and hydrogen atoms were added. Chain B was extracted from PDB file 1IC8 and saved as the structure of the transcription domain of HNF-1α as only one unit of the domain was needed. The dimerization domain of HNF-1α exists as a homodimer in cells, therefore, chain B of PDB file 1G39 was saved as a single unit of the dimerization domain in order to compare interactions between the dimerization domain as a homodimer and as a single unit. Chains A and C from PDB file 1G39 were extracted and saved as the structure of a homodimer of the dimerization domain of HNF-1α. Each structural file was opened in AutoDockTools and residues SER19, GLY20, LEU21, and SER22 in the dimerization domain of HNF-1α as both a homodimer and a single unit were selected as flexible residues. Flexible and rigid structural files were saved for both the dimerization domain as a homodimer and single unit, but only a rigid structural file of the transcription domain was saved given its lack of flexibility [1].

#### Molecular docking

The rigid structural file of each HNF-1α structure was opened in AutoDockTools. A grid box that encapsulated each structure, but that was not of excessive size, was created with dimensions set to one angstrom. The dimensions of the grid box for the transcription domain (Center X = 15.618, Center Y = -15.072, Center Z = 3.754, Size X = 52, Size Y = 56, Size Z = 68), dimerization domain as a homodimer (Center X = 9.05, Center Y = -20.995, Center Z = 10.324, Size X = 40, Size Y = 40, Size Z = 50), and as a single unit (Center X = -4.864, Center Y = -24.565, Center Z = 12.326, Size X = 28, Size Y = 32, Size Z = 24) were saved in configuration files. Molecular docking experiments were conducted using AutoDock Vina [21] using the configuration files created. An exhaustiveness value of 200 was used for each docking run of the dimerization domain as a homodimer and as a single unit, but an exhaustiveness value of 250 was used for DNA binding domain due to its larger size. The exhaustiveness value of each run was proportionate to the size of the search space for each structure, and is proportionate to the number of starting ligand poses. Therefore, a larger exhaustiveness value decreases the chances of not finding the best binding conformation for each ligand. The resulting ligand binding conformations were analyzed using DS 4.0. The best conformation of each ligand was determined as the conformation that had the highest binding affinity for each HNF-1α structure.

#### Validation of molecular docking methodology

AutoDock Vina has already been used to analyze the interactions of phenolic compounds before [22], but has not been used to analyze any of the phenolic compounds studied with the computational methodology used. In order to test the efficiency of the molecular docking scheme, the hepatocyte nuclear factor-4α (HNF-4α) PDB file 4IQR with myristic acid as a ligand was used as no HNF-1α structure has been solved that includes a docked ligand to this date. The protein chains A and I in PDB 4IQR were extracted and saved as a PDB structural file using DS 4.0, and ligand chain A (myristic acid) was also extracted. Protein chain A and ligand chain A were used as they interact with each other, while protein chain I was kept in the structural file due to its close interaction with protein chain A. The receptor file was opened in AutoDockTools and saved as a rigid structural file. The extracted myristic acid ligand was added hydrogen atoms and applied the CHARMM22 and DREIDING force fields using DS 4.0. The myristic acid ligand was then opened in AutoDockTools and added Gasteiger charges. All rotatable bonds detected by AutoDockTools for the ligand were kept. The rigid PDBQT receptor file was opened in AutoDockTools and had a grid encapsulating it that had the same size dimensions as those used for the transcription domain receptor file of HNF-1α set to one angstrom. The dimensions of the grid search space (Center X = 33.187, Center Y = -8.81, Center Z = 29.698, Size X = 52, Size Y = 56, Size Z = 68) were saved in a configuration file that had an exhaustiveness value of 250 like the HNF-1α transcription domain docking runs. The search space parameters of the transcription domain were used as they were the largest. AutoDock Vina was used to perform the molecular docking runs with the configuration file created. The resulting myristic acid conformation with the highest binding affinity was compared to the conformation in the original crystal structure in PDB file 4IQR using the UCSF Chimera package (version 1.9) [23]. Chimera was developed by the Resource for Biocomputing, Visualization, and Informatics at the University of California, San Francisco (NIGMS grant P41-GM103311). Amino acid interactions between myristic acid conformations were analyzed using DS 4.0.

### Western Blotting Materials

Human Caco-2 cells (HTB-37), human normal colon CCD-33Co cells (CRL-1539), Eagle’s Minimum Essential Medium, and 0.25% (w/v) trypsin-0.53 mM EDTA were purchased from American Type Culture Collection (ATCC) (Manassas, VA, USA). Penicillin-streptomycin was purchased from Corning Inc. (Corning, NY, USA), fetal bovine serum (FBS) from Hyclone (Thermo Scientific Hyclone, Logan, UT, USA). VitaBlue 20% anthocyanin blueberry extract (N208.2) was provided by FutureCeuticals, Inc. (Momence, IL, USA). Primary mouse monoclonal antibody GADPH (sc-47724), primary rabbit polyclonal antibody GAPDH (sc-25778), primary goat polyclonal antibody HNF-1α (N-19) (sc-6548), donkey polyclonal anti-goat IgG horseradish conjugate secondary antibody (sc-2020), purified donkey polyclonal anti-goat IgG horseradish conjugate secondary antibody (sc-2056), and purified goat polyclonal anti-rabbit IgG horseradish conjugate secondary antibody (sc-2054)were purchased from Santa Cruz Biotechnology, Inc. (Santa Cruz, CA). Sheep polyclonal anti-mouse IgG horseradish conjugate secondary antibody (NXA931) was purchased from GE Healthcare Biosciences (Pittsburg, PA). All other chemicals were purchased from Sigma-Aldrich unless otherwise stated.

### Preparation of Anthocyanin Extracts from Blueberry and Blackberry Fermented Beverages

Anthocyanin (ANC) extracts from fermented blueberry and blackberry beverages were prepared as described previously [19]. In short, highbush blueberries (*Vaccinium corymbosum*) and blackberry cultivars (*Rubus fruticosus*) were collected from Dixon Springs Agricultural Center in Simpson, Illinois during the ripening season of 2010. The collected blueberries were frozen and crushed to produce blueberry juice which was fermented with *Saccharomyces bayanus* at 23°C. Dry wine yeast Lavin EC 1118 (*S*. *bayanus*) was obtained from Presque Isle Wine Cellars (North East, PA). Collected blackberries were fermented in a similar manner. Room temperature blueberry and blackberry wines were mixed to create a 50% blackberry: 50% blueberry wine mixture. For this study, 100% blackberry wine was also used along with the mixture created. Selected wines were acidified with 0.1% TFA, dealcoholized using rotoevaporation that did not exceed 40°C, and then mixed with preconditioned amberlite XAD-7 resin to remove impurities. Resulting sample mixtures were loaded on a column (30 X 3 cm) with the amberlite XAD-7 resin and washed with acidified methanol (0.1% TFA) to elute polyphenols from the amberlite resin. Polyphenolic extracts were concentrated using rotoevaporation that did not exceed 40°C, and were then reconstituted with an acidified water:methanol (80:20; 0.1% TFA) solution. The solution was loaded on a Sephadex LH-20 column (30 X 3 cm) preconditioned with acidified water:methanol (80:20; 0.1% TFA). Using an isocratic elution (80:20; 0.1% TFA), fractions were collected at the moment colored material began to elute to generate ANC fractions of each original wine mixture. Rotoevaporation was used to remove the solvent of the ANC extract mixtures. The resulting extracts were freeze-dried and stored at -80°C until further use.

### Cell Culture and Nuclear Extraction

Cells were plated at a density of 2 x 10^5^ cells/well and allowed to grow for 24 h at 37°C. The cells were then treated with 5 or 50 μM cyanidin-3-*O*-glucoside equivalents of VitaBlue commercial ANC blueberry extract, ANC extract from 50% blackberry: 50% blueberry dealcoholized wine, or ANC extract from 100% blackberry dealcoholized wine, or 5 μM cyandin-3-*O*-glucoside (C3G, ≥ 99%) or 5 μM delphinidin-3-*O*-glucoside (D3G, ≥ 97%) for 24 h. After treatment, the cells were harvested by washing with 1 mL PBS followed by washing with trypsin-EDTA solution (1 mL, twice), and plates were incubated at 37°C for 5–10 min. Cell lysates were collected with 1 mL PBS and pelleted by centrifugation at 500 x g for 10 min, and the supernatant was discarded.

Separation of nuclear extract was conducted using NE-PER nuclear and cytoplasmic extraction kit (Thermo Scientific, Waltham, MA). After centrifugation, cell pellets were treated with 150 μL of ice-cold cytoplasmic extraction reagent I, vortexed for 15 s, and incubated on ice for 10 min. Ice-cold cytoplasmic extraction reagent II (8.25 μL) containing 0.5 M EDTA solution (1:100 ratio) was then added, tubes were vortexed for 5 s and then incubated on ice for 1 min. After incubation, the tube was vortexed for 5 s and centrifuged at 16,000 x g for 5 min at 4°C. The supernatant (cytoplasmic extract) was immediately removed, and the remaining pellet was then resuspended in 75 μL ice-cold nuclear extraction reagent, vortexed for 15 sec and incubated on ice for 40 min with 15 s vortexing every 10 min. The mixture was centrifuged at 16,000 x g for 10 min at 4°C, and the supernatant (nuclear extract) was collected in clean pre-chilled tubes. Protein concentrations of the nuclear extracts were determined using the DC Protein Assay (Bio-Rad Laboratories, Inc., Hercules, CA). Laemmli buffer containing 5% β-mercaptoethanol was added to the nuclear extracts (1:1). The samples were boiled for 5 min, and then stored at -80°C until use.

### Western Blotting Analysis

Ten μg of nuclear protein/well was loaded in 4–20% gradient SDS-polyacrylamide gels (Bio-Rad Laboratories, Inc.). The separated proteins were transferred to PVDF membrane and blocked with 3% non-fat dry milk in 0.1% TBST for 1 h at room temperature if they were from Caco-2 cells, and 5% non-fat dry milk in 0.1% TBST if they were from normal colon cells. After blocking, the membranes were washed with 0.1% TBST (5 times, 5 min each) and incubated with primary antibodies (1:500) overnight at 4°C. Nuclear protein extracts from normal colon cells had their appropriate horseradish peroxidase conjugate secondary antibody co-incubated with the primary antibody overnight in order to reduce protein degradation. The membranes were washed again and incubated with anti-mouse or anti-goat IgG horseradish peroxidase conjugate secondary antibodies (1:2500) for 1 h at room temperature if they had not been incubated with a secondary antibody. After incubation and repeated washing, the membranes were prepared for detection using a 1:1 mixture of chemiluminescent reagents A (luminol solution) and B (peroxide solution) (GE Healthcare Biosciences). The membrane pictures were taken on a GelLogic 4000 Pro Imaging System (Carestream Health, Inc., Rochester, NY). The relative amount of each target protein was normalized to glyceraldehyde 3-phosphate dehydrogenase (GAPDH).

### Statistics

Data were expressed as means of independent duplicates ± SEM with at least three replicates. Statistical analysis was performed using statistical program R (version 3.1.0) [24]. Group mean comparisons were conducted using a Games-Howell post hoc test and considered significant at P < 0.05 after a one-way analysis of variance (ANOVA) with α = 0.05. Outliers were determined and discarded using the Median Absolute Deviation (MAD) method with a constant of 1.4826 correlated to normality and a threshold of ± 2 times the MAD from the median as described before [25].

## Results

### Delphinidin-3-*O*-Arabinoside had the Highest Affinity for the Transcription Domain of HNF-1α

**Fig 2:**
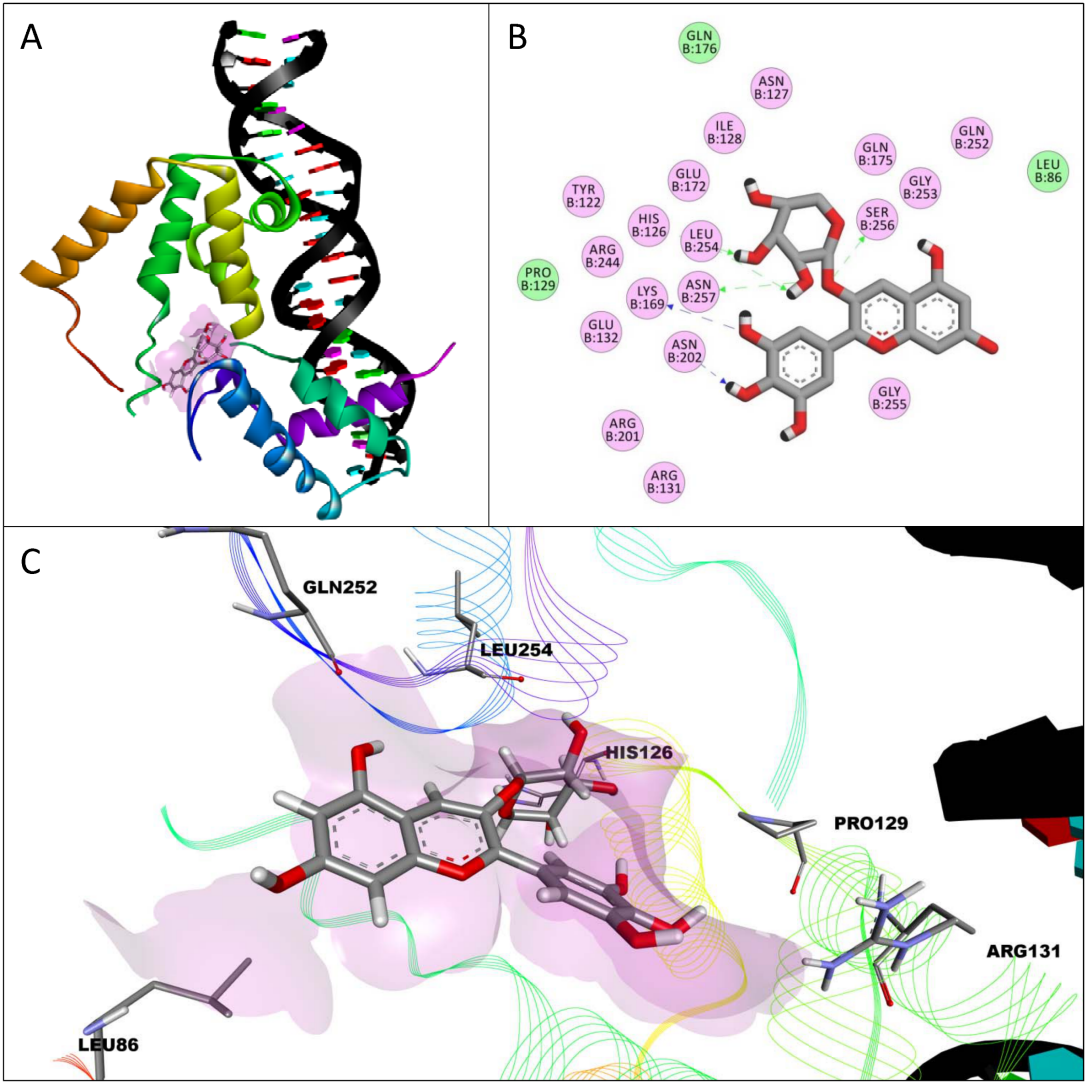
Interactions between delphinidin-3-*O*-arabinoside and the transcription domain of hepatocyte nuclear factor-1α (HNF-1α). The conformation of delphinidin-3-*O*-arabinoside with the highest binding affinity for the transcription domain of HNF-1α attached to a DNA construct from PDB file 1IC8 is shown (A) with a lilac surface representing a general surface of the domain (A, C). Amino acids in the transcription domain of HNF-1α had electrostatic (pink) and van der Waals (green) interactions with delphinidin-3-O-arabinoside (B). Hydrogen bonds are shown as one headed, dashed arrows formed with amino acid main-chain (green) or side-chain (blue) atoms. A 3-dimensional representation of delphinidin-3-*O*-arabinoside interacting with the transcription domain of HNF-1α is shown (C) with certain interacting amino acids highlighted and labeled to show their relative position.

The interactions for each compound with the highest binding affinity for the transcription domain of HNF-1α are shown in Table 1. Delphinidin-3-*O*-arabinoside had the highest binding affinity of all compounds (-8.3 kcal/mol) studied and interacted with the transcription domain of HNF-1α on the opposite face from where the domain binds to DNA (Fig 2A). The interactions of delphinidin-3-*O*-arabinoside with HNF-1α (Fig 2B) and the conformation (Fig 2C) are shown for reference. The top three compounds with the highest binding affinity for the transcription domain of HNF-1α were all anthocyanins that had electrostatic interactions with the amino acids ASN127 and ASN257. Flavone and hesperetin were the only two compounds to have no interactions with the amino acid ASN257, but all tested compound interacted with the amino acid ILE128. Malvidin-3-*O*-arabinoside and delphinidin-3-*O*-arabinoside interacted in common with 19 amino acids (HIS126, ASN127, ILE128, PRO129, ARG131, GLU132, LYS169, GLU172, GLN175, GLN176, ARG201, ASN202, ARG244, GLN252, GLY253, LEU254, GLY255, SER256, ASN257) and 13 of these interactions were similar (HIS126, ASN127, PRO129, ARG131, GLU132, LYS169, GLN175, ASN202, ARG244, GLN252, GLY253, LEU254, SER256, ASN257). Both of these conformations had the most hydrogen bonds (6) with the amino acids of the transcription domain of HNF-1α. Delphinidin-3-*O*-arabinoside had a van der Waals interaction with the amino acid LEU86 which was seen only by another anthocyanidin, malvidin. Malvidin-3-*O*-galactoside and malvidin-3-*O*-glucoside were the only compounds that interacted with the amino acid ARG171, while malvidin was the only compound to interact with the amino acid HIS179 through van der Waals interactions. Apigenin and luteolin were the only compounds to interact with PHE204. Flavone had the most van der Waals interactions of any other of the compounds tested for the transcription domain. Cyanidin-3-*O*-glucoside and hesperetin had interactions in common with 13 amino acids (LYS120, LEU123, GLN124, ASN127, ILE128, GLN130, LEU148, ASN149, LYS205, TRP206, GLY207, PRO208, GLN211) in which nine were similar (LYS120, GLN124, ASN127, ILE128, GLN130, ASN149, TRP206, PRO208, GLN211). All the flavonoids tested had higher affinity for the transcription domain of HNF-1α than the stilbenoid resveratrol.

**Table 1:** Interactions of each compound’s conformation with the highest binding affinity with the transcription domain of HNF-1α.

Compound	PubChem ID	Binding Affinity (kcal/mol)	Total Amino Acid Interactions	Amino Acids with Electrostatic Interactions	Amino Acids with van der Waals Interactions	Total Number of H-bonds	π-interactions
Delphinidin-3-*O*-Arabinoside	25087690	-8.3	21	TYR122, HIS126, ASN127, ILE128, ARG131, GLU132, LYS169, GLU172, GLN175, ARG201, ASN202, ARG244, GLN252, GLY253, LEU254, GLY255, SER256, ASN257	LEU86, PRO129, GLN176	6	-
Malvidin-3-*O*-Arabinoside	25079994	-8.0	19	HIS126, ASN127, ARG131, GLU132, LYS169, GLN175, GLN176, ASN202, ARG244, GLN252, GLY253, LEU254, SER256, ASN257	ILE128, PRO129, GLU172, ARG201, GLY255	6	π-cation with LYS169 & ARG201
Cyanidin-3-*O*-Glucoside	197081	-7.8	16	GLN124, ASN127, ILE128, PRO129, GLN130, LEU148, ASN149, ARG203, TRP206, GLY207, PRO208, GLN211, ASN257	LYS120, LEU123, LYS205	1	π-cation with LYS205, π-σ with GLN124
Delphinidin	128853	-7.8	16	HIS126, ASN127, PRO129, ARG131, GLU132, LYS169, GLN175, GLN176, ASN202, ARG244, GLY253, LEU254, SER256, ASN257	ILE128, GLU172	4	π-cation with LYS169
Cyanidin	128861	-7.7	14	HIS126, LYS169, GLU172, GLU175, GLN176, ARG201, ASN202, ARG244, LEU254, SER256, ASN257	TYR122, ILE128, GLY255	5	-
Luteolin	5280445	-7.7	13	LYS120, LEU123, GLN124, ASN127, ILE128, PRO129, GLN130, ASN149, ARG203, PHE204, LYS205, ASN257	LEU148	1	π-cation with LYS205
Apigenin	5280443	-7.4	13	LYS120, GLN124, ILE128, GLN130, ASN149, ARG203, PHE204, LYS205, ASN257	LEU123, ASN127, PRO129, LEU148	2	π-cation with LYS205
Malvidin-3-*O*-Galactoside	94409	-7.4	16	TYR122, HIS126, LYS169, GLU172, GLN175, ARG201, ASN202, ARG244, LEU254, GLY255, SER256, ASN257	ILE128, ARG131, GLU132, ARG171	5	-
Malvidin-3-*O*-Glucoside	443652	-7.4	17	TYR122, HIS126, LYS169, GLU172, GLN175, ARG201, ASN202, ARG244, GLY253, LEU254, SER256, ASN257	ILE128, ARG131, GLU132, ARG171, GLY255	5	-
Naringenin	932	-7.4	17	HIS126, ARG131, GLU132, LYS169, GLU172, GLN176, ARG201, ASN202, ARG244, SER256	TYR122, ILE128, GLN175, GLY253, LEU254, GLY255, ASN257	2	π-cation with ARG131
Flavone	10680	-7.3	16	LYS169, SER256	TYR122, HIS126, ILE128, ARG131, GLY132, GLU172, GLN175, GLN176, ARG201, ASN202, ARG244, GLY253, LEU254, GLY255	1	-
Hesperetin	72281	-7.2	13	LEU123, GLN124, ASN127, ILE128, GLN130, ASN149, LYS205, TRP206, PRO208, GLN211	LYS120, LEU148, GLY207	3	π-cation with LYS120, π-σ with PRO208
Malvidin	159287	-6.9	19	TYR122, HIS126, ASN127, ILE128, LYS169, GLU172, GLU175, GLN176, ARG201, ASN202, ARG244, GLY253, SER256, ASN257	LEU86, PRO129, HIS179, GLN252, GLY255	4	π-cation with LYS169 & ARG244
Resveratrol	445154	-6.9	16	HIS126, ARG131, GLU132, LYS169, GLN176, ARG201, ASN202, ARG244	TYR122, ILE128, GLU172, GLY253, LEU254, GLY255, SER256, ASN257	2	π-cation with ARG131

### Anthocyanins and Anthocyanidins Interacted Most with a Homodimer of the Dimerization Domain of HNF-1α

**Fig 3:**
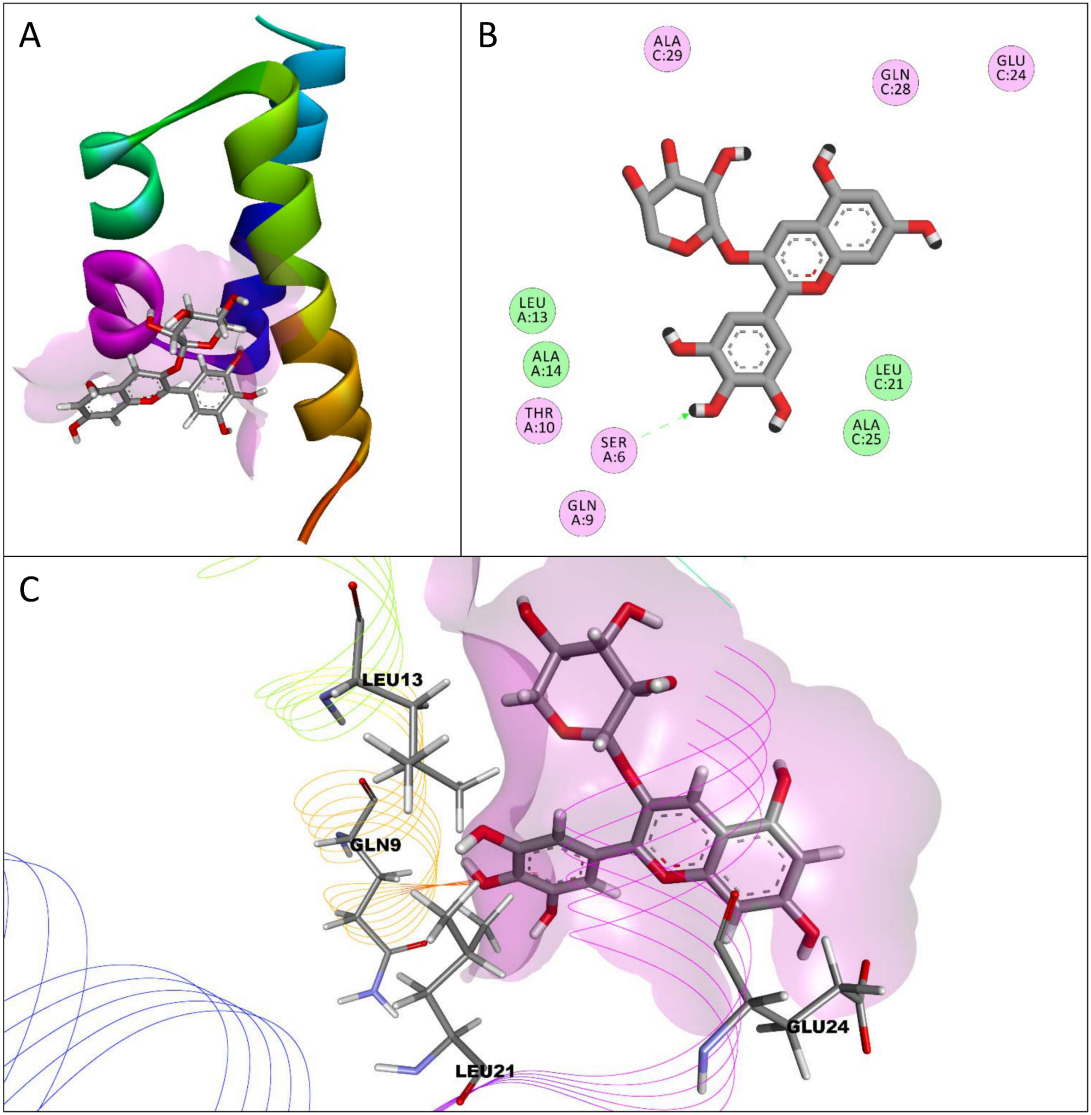
Interactions between delphinidin-3-*O*-arabinoside and a homodimer of the dimerization domain of hepatocyte nuclear factor-1α (HNF-1α). The conformation of delphinidin-3-*O*-arabinoside with the highest binding affinity for the dimerization domain of HNF-1α as a homodimer is shown (A) with a lilac surface representing a general surface of the homodimer (A, C). Amino acids in the homodimer of the dimerization domain of HNF-1α had electrostatic (pink) and van der Waals (green) interactions with delphinidin-3-*O*-arabinoside (B). A hydrogen bond between atoms in the main-chain of SER6 and delphinidin-3-*O*-arabinoside is represented by a one headed, dashed green arrow. A 3-dimensional representation of delphinidin-3-*O*-arabinoside interacting with the dimerization domain of HNF-1α as a homodimer is shown (C) with certain interacting amino acids highlighted and labeled to show their relative position.

The interactions between the conformations with the highest binding affinity of each compound and the dimerization domain of HNF-1α as a homodimer are shown in Table 2. Delphinidin-3-*O*-arabinoside had the highest binding affinity (-7.2 kcal/mol) for the homodimer of the dimerization domain. Interactions and the conformation of delphinidin-3-*O*-arabinoside are shown in Fig 3. Delphinidin-3-*O*-arabinoside did not have the most interactions with the structure of HNF-1α, instead malvidin, malvidin-3-*O*-glucoside, delphinidin, and cyanidin did. All the compounds tested had electrostatic interactions with the amino acids SER6 and GLN9, except for flavone, which had van der Waals interactions with these two amino acids. Every compound had an electrostatic interaction with the amino acid THR10 and a van der Waals interaction with amino acid LEU13. Cyanidin-3-*O*-glucoside was the only compound to have an electrostatic interaction with amino acid LEU21, even though every other compound had a van der Waals interaction with this amino acid. Apigenin and resveratrol were the only compounds that interacted with the amino acid LEU17, and delphinidin and malvidin were the only compounds to interact with amino acid LEU5. Cyanidin was the only compound that interacted with amino acid LEU26, and flavone had the most van der Waals interactions with the homodimer. Delphinidin-3-*O*-arabinoside only had one hydrogen bond despite having the highest affinity, while malvidin-3-*O*-arabinoside had the most hydrogen bonds (6). Hesperetin, cyanidin, and delphinidin were the only compounds to have π-interactions. All the flavonoids tested had higher binding affinities than the stilbenoid resveratrol, similar to the transcription domain molecular dockings.

**Table 2:** Interactions of each compound’s conformation with the highest binding affinity with a homodimer of the dimerization domain of HNF-1α.

Compound	PubChem ID	Binding Affinity (kcal/mol)	Total Amino Acid Interactions	Amino Acids with Electrostatic Interactions	Amino Acids with van der Waals Interactions	Total Number of H-bonds	π-interactions
Delphinidin-3-*O*-Arabinoside	25087690	-7.2	10	SER6, GLN9, THR10, GLU24, GLN28, ALA29	LEU13, ALA14, LEU21, ALA25	1	-
Cyanidin	128861	-7.0	11	SER6, GLN9, THR10, GLY20, SER22, ALA25, LEU26,GLN28, ALA29	LEU13, LEU21	4	π-σ with LEU21
Delphinidin	128853	-6.9	11	SER6, GLN9, THR10, GLY20, SER22, ALA25, ALA29	LEU5, LEU13, LEU21, GLN28	4	π-σ with LEU21
Malvidin-3-*O*-Arabinoside	25079994	-6.9	10	SER6, GLN9, THR10, SER22, GLU24, ALA25, GLN28, ALA29	LEU13, LEU21	6	-
Cyanidin-3-*O*-Glucoside	197081	-6.8	9	SER6, GLN9, THR10, GLY20, LEU21, SER22, GLU24	LEU13, ALA25	5	-
Apigenin	5280443	-6.6	9	SER6, GLN9 THR10	LEU13, ALA14, LEU17, LEU21, ALA25, GLN28	1	-
Hesperetin	72281	-6.6	9	SER6, GLN9, THR10, ALA14	LEU13, LEU21, ALA25, GLN28, ALA29	1	π-σ with THR10
Luteolin	5280445	-6.6	9	SER6, GLN9, THR10, GLU24, GLN28	LEU13, LEU21, ALA25, ALA29	2	-
Naringenin	932	-6.6	8	SER6, GLN9, THR10, ALA14	LEU13, LEU21, ALA25, ALA29	-	-
Flavone	10680	-6.4	9	THR10	SER6, GLN9, LEU13, ALA14, LEU21, ALA25, GLN28, ALA29	-	-
Malvidin	159287	-6.4	11	SER6, GLN9, THR10, ALA25, ALA29	LEU5, LEU13, ALA14, LEU21, SER22, GLN28	3	-
Malvidin-3-*O*-Galactoside	94409	-6.4	10	SER6,GLN9, THR10, ALA14, GLU24, GLN28, ALA29	LEU13, LEU21, ALA25	1	-
Malvidin-3-*O*-Glucoside	443652	-6.4	11	SER6, GLN9, THR10, GLY20, SER22, GLU24, GLN28	LEU13, LEU21, ALA25, ALA29	5	-
Resveratrol	445154	-6.0	9	SER6, GLN9, THR10	LEU13, ALA14, LEU17, LEU21, ALA25, GLN28	-	-

### Flavone had the Highest Binding Affinity for a Single Unit of the Dimerization Domain of HNF-1α

**Fig 4:**
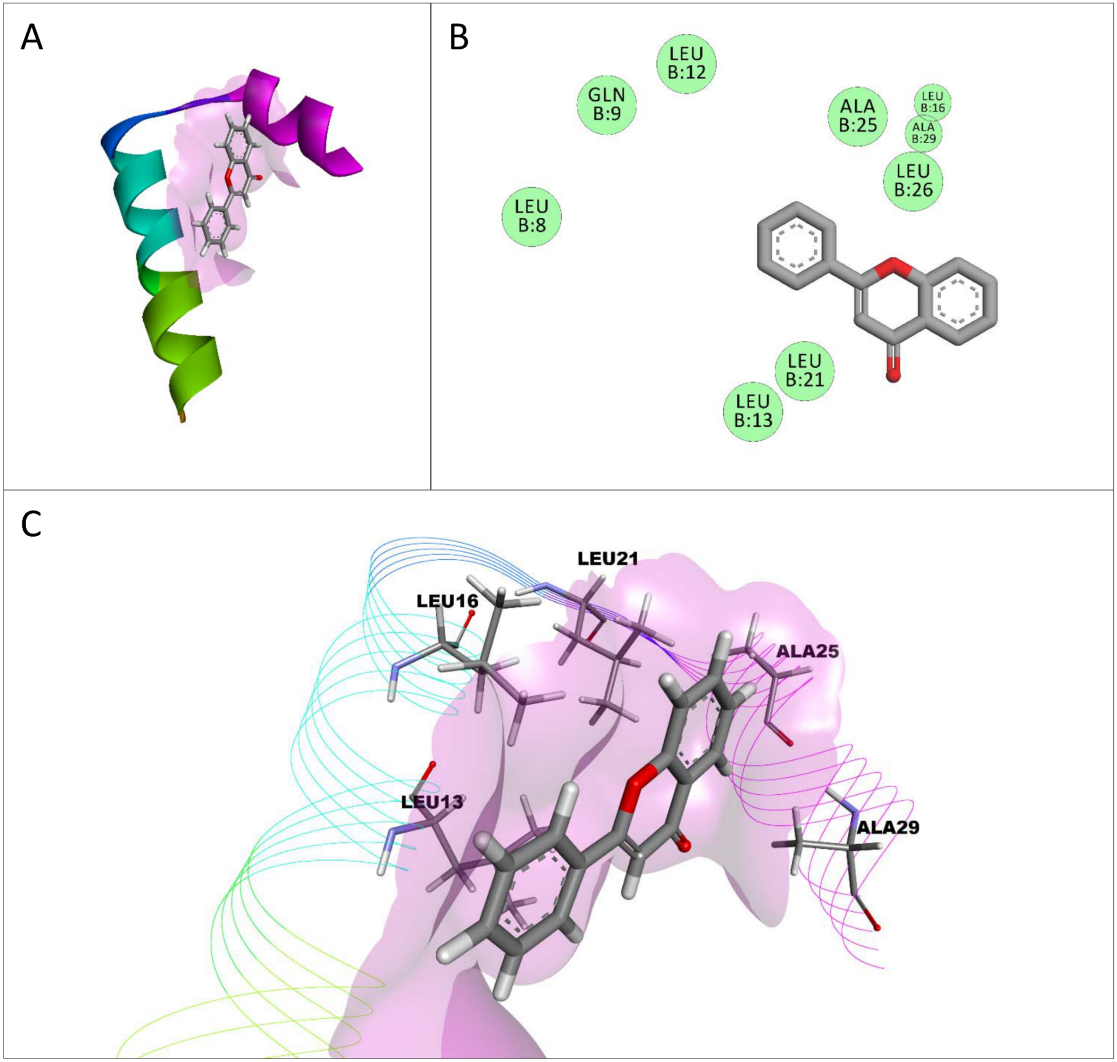
Interactions between flavone and the dimerization domain of hepatocyte nuclear factor-1α (HNF-1α). The conformation of flavone with the highest affinity for a single unit of the dimerization domain of HNF-1α is shown (A) with a lilac surface representing a general surface of the dimerization domain of HNF-1α (A, C). Amino acids in the dimerization domain of HNF-1α only had van der Waals (green) interactions with flavone (B). A 3-dimensional representation of flavone interacting with the dimerization domain of HNF-1α is shown (C) with certain interacting amino acids highlighted and labeled to show their relative position.

The binding affinities and interactions of each conformation, with the highest binding affinity for each compound with a single unit of the dimerization domain of HNF-1α, are shown in Table 3. Flavone had the highest binding affinity (-6.5 kcal/mol) with the dimerization domain of HNF-1α, even though it had only van der Waals interactions (Fig 4). The conformation and placement of flavone with this domain are also shown in Fig 4. Apigenin and flavone had interactions with eight amino acids in common (GLN9, LEU12, LEU13, LEU16, LEU21, ALA25, LEU26, ALA29), in which six of those interactions were the same with the same amino acids (LEU12, LEU16, LEU21, ALA25, LEU26, ALA29). Malvidin-3-*O*-glucoside had the most interactions with the dimerization domain (12), while cyanidin-3-*O*-glucoside had the largest number of hydrogen bonds (3). All the compounds tested had interactions with amino acids GLN9 and LEU13 except for hesperetin, which was the only compound which had interactions with amino acids LYS4 and ALA15. Malvidin-3-*O*-galactoside was the only compound to have an interaction with amino acid VAL2 and not with amino acid LEU12, and along with hesperetin, it was one of two compounds to interact with amino acid GLN7. Hesperetin was the only ligand to interact with amino acid GLU11. Furthermore, hesperetin and malvidin-3-*O*-glucoside were the only two ligands to interact with the amino acid LEU5. Luteolin, cyanidin, resveratrol, and delphinidin all had a π-σ interaction with the amino acid LEU13. The anthocyanidin malvidin had the lowest binding affinity for this domain.

**Table 3:** Interactions of each compound’s conformation with the highest binding affinity with a single unit of the dimerization domain of HNF-1α.

Compound	PubChem ID	Binding Affinity (kcal/mol)	Total Amino Acid Interactions	Amino Acids with Electrostatic Interactions	Amino Acids with van der Waals Interactions	Total Number of H-bonds	π-interactions
Flavone	10680	-6.5	9	-	LEU8, GLN9, LEU12, LEU13, LEU16, LEU21, ALA25, LEU26, ALA29	-	-
Malvidin-3-*O*-Glucoside	443652	-6.2	12	SER6, GLN9, THR10, ALA25, LEU26, ALA29	LEU5, LEU8, LEU12, LEU13, LEU16, LEU21	1	-
Malvidin-3-*O*-Arabinoside	25079994	-6.1	8	SER6, GLN9, THR10, LEU26	LEU12, LEU13, LEU16, ALA29	2	-
Apigenin	5280443	-5.9	8	GLN9, LEU13	LEU12, LEU16, LEU21, ALA25, LEU26, ALA29	-	-
Luteolin	5280445	-5.8	8	SER6, GLN9, THR10	LEU12, LEU13, LEU16, LEU26, ALA29	2	π-σ with LEU13
Naringenin	932	-5.8	5	GLN9, THR10	LEU12, LEU13, LEU16	1	-
Cyanidin-3-*O*-Glucoside	197081	-5.7	5	SER6, GLN9, THR10	LEU12, LEU13	3	-
Delphinidin-3-*O*-Arabinoside	25087690	-5.6	7	SER6, GLN9, THR10	LEU12, LEU13, ALA14, LEU16	2	π-σ with GLN9
Cyanidin	128861	-5.6	6	SER6, GLN9, THR10	LEU12, LEU13, LEU16	1	π-σ with LEU13
Hesperetin	72281	-5.6	7	LYS4, GLN7, LEU8, GLU11, LEU12	LEU5, ALA15	1	π-cation with LYS4
Resveratrol	445154	-5.6	7	SER6, GLN9, THR10	LEU12, LEU13, LEU16, LEU21	-	π-σ with LEU13
Delphinidin	128853	-5.5	7	SER6, GLN9, THR10, LEU13	LEU12, LEU16, ALA29	1	π-σ with LEU13
Malvidin-3-*O*-Galactoside	94409	-5.5	8	SER6, GLN7, GLN9, THR10, ALA14	VAL2, LEU13, LEU17	2	π-σ with THR10
Malvidin	159287	-5.2	6	GLN9, THR10	LEU12, LEU13, ALA14, LEU17	1	-

### Computational Methodology Gave Similar Conformation to Crystal Structure

**Fig 5:**
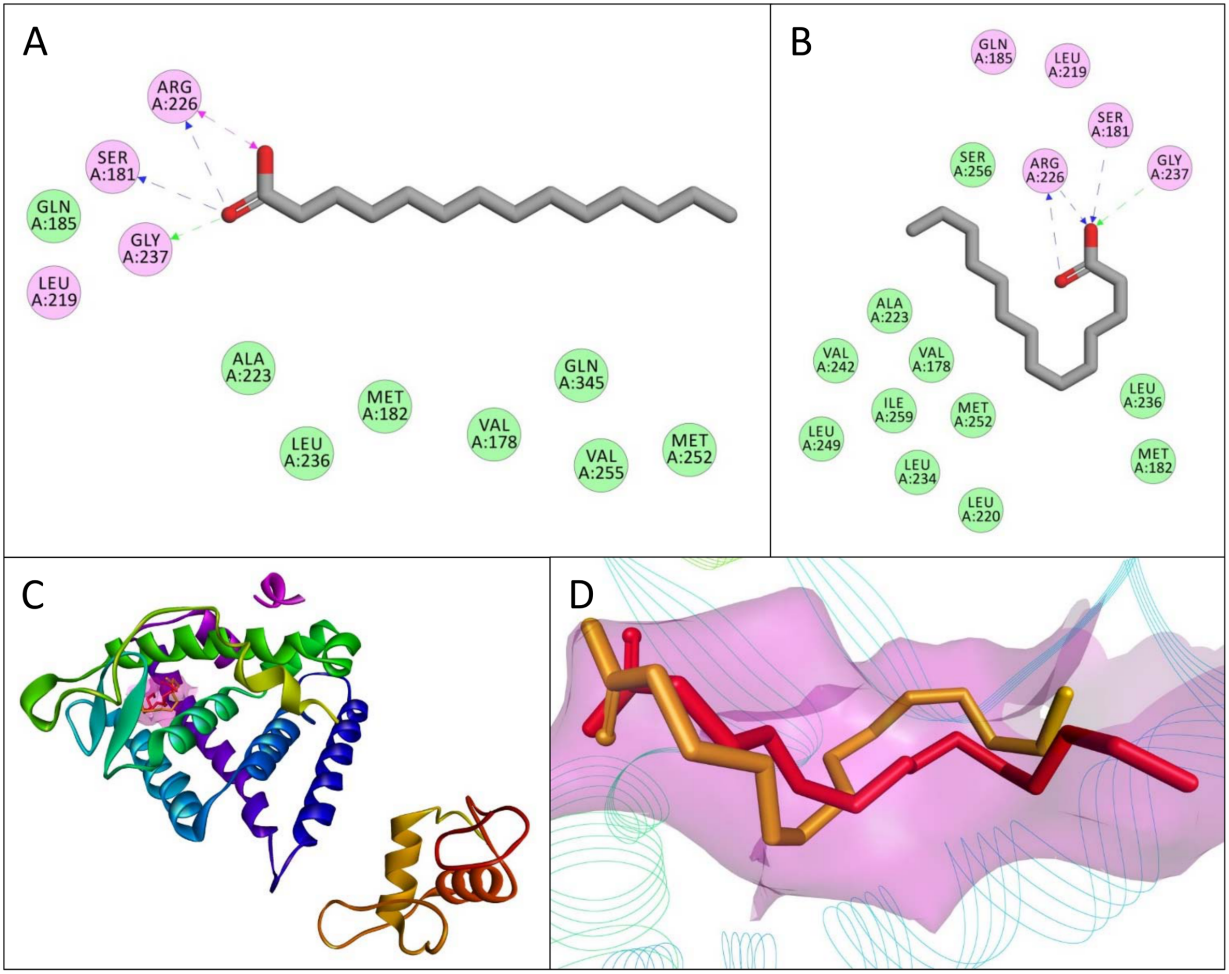
Validation study by re-docking myristic acid to hepatocyte nuclear factor-4α (HNF-4α). The interactions that myristic acid has with HNF-4α in PDB file 4IQR are shown (A) alongside the interactions the best experimental docking conformation of myristic acid had with the same structure (B). Amino acids from HNF-4α that interacted with myristic acid in each conformation either had electrostatic (pink) or van der Waals (green) interactions. Hydrogen bonds are shown as one headed, dashed arrows formed with amino acid main-chain (green) or side-chain (blue) atoms, while pink double headed, dashed arrows represent charged interactions. The area of interaction both myristic acid conformations had with HNF-4α (C) is shown alongside a comparison of both the experimental conformation (orange) and the conformation (red) found in PDB file 4IQR (D). A lilac surface around both conformations (C, D) represents a general surface of HNF-4α.

The resulting root-mean-square deviation (RMSD) of all heavy atoms in the conformation of myristic acid in the hepatocyte nuclear factor-4α (HNF-4α) crystal structure of The RCSB Protein Data Bank (PDB) file 4IQR, compared to the resulting myristic acid experimental conformation from AutoDock Vina, was 1.458 angstroms using the match function from the USFC Chimera package. Both conformations had nine exact amino acid interactions (Fig 5A and 5B) (VAL178, SER181, MET182, LEU219, ALA223, ARG226, LEU236, GLY237, MET252) within the same location of HNF-4α (Fig 5C) given the large search space parameters. The conformations can be compared visually in Fig 5D. Both conformations show myristic acid having hydrogen bonds with HNF-4α amino acids SER181, ARG226, and GLY237 (Fig 5A and 5B). The charged interaction between ARG226 and myristic acid in PDB file 4IQR is not observed in the experimental conformation from AutoDock Vina, instead a second hydrogen bond with ARG226 is observed. A van der Waals interaction with GLN185 is observed in PDB file 4IQR while an electrostatic interaction with the same amino acid resulted in the experimental conformation. Given the similarity between the known and experimental conformations of myristic acid, the computational methodology used showed to produce conformations similar to those found in known structures, and was therefore used in this study.

### Anthocyanin Mixtures Increased Expression of Hepatocyte Nuclear Factor-1α

**Fig 6:**
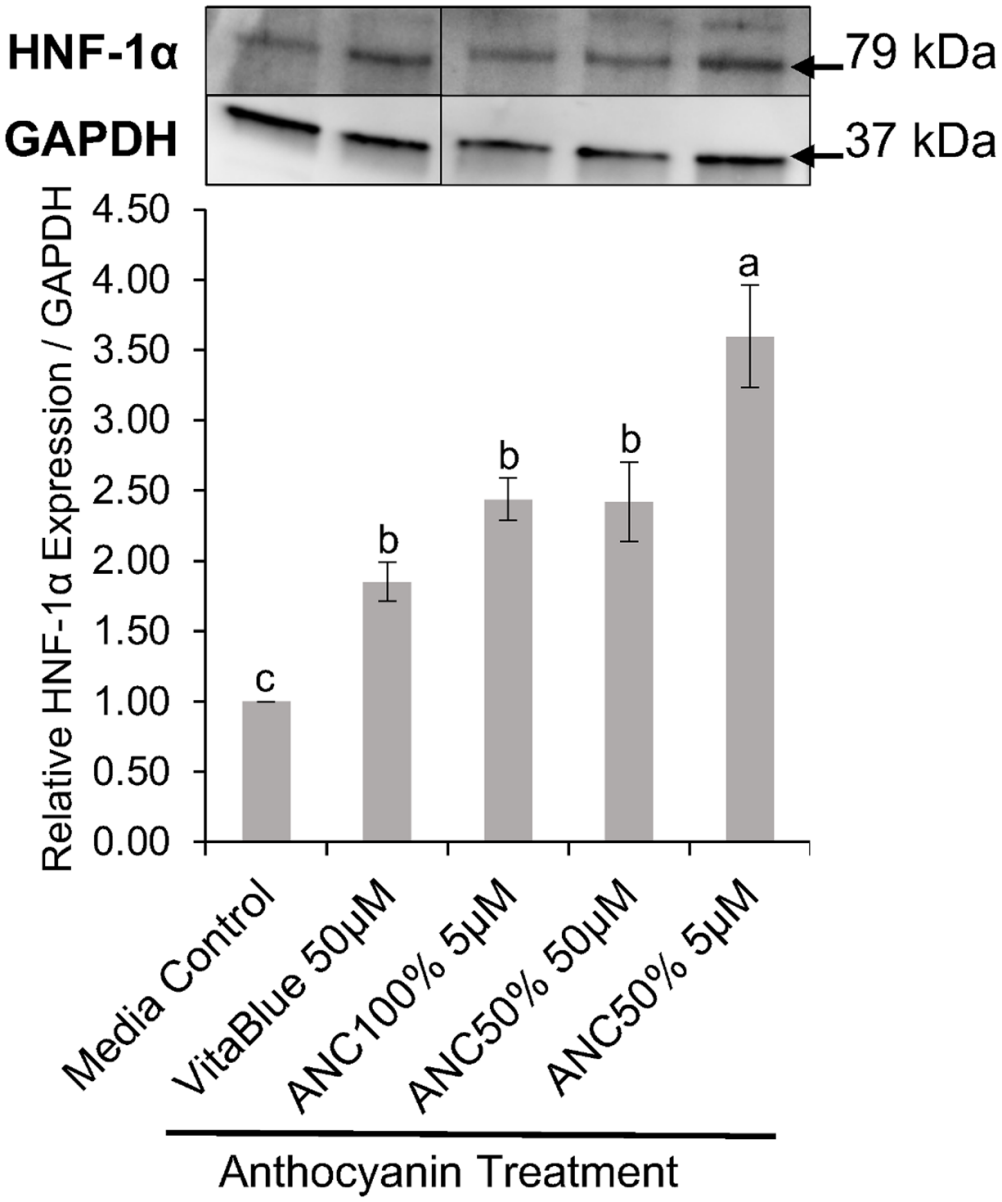
Effects of anthocyanin extracts on nuclear hepatocyte nuclear factor-1α (HNF-1α) expression in Caco-2 cells. Anthocyanin extract treatments from blueberry and blackberry fermented beverages are named as ANC with the percentage of the beverage mixture that was derived from blackberries (ANC50% = 50% blueberry: 50% blackberry, ANC100% = 100% blackberry). VitaBlue is a commercial blueberry 20% anthocyanin extract. Dosage (μM) is given in cyanidin-3-O-glucoside equivalents, and the media control was from Caco-2 cells grown only in media. Relative expression of nuclear HNF-1α over GAPDH is graphed with the mean ± SEM with different letters indicating significantly different expression values (n ≥ 3, P < 0.05). Western blot bands for HNF-1α (79 kDa) and GAPDH (37 kDa) are displayed directly above the respective column of each treatment or control. Bands within the same membrane that were not quantified were cropped out for clarity (See supplementary S1 Fig for full membrane images). Thin bands above the HNF-1α bands are considered to be from non-specific binding of the HNF-1α polyclonal antibody.

HNF-1α expression was up-regulated in the nucleus of Caco-2 cells by all the anthocyanin (ANC) containing extracts used in Western blotting (Fig 6). The 5 μM (cyanidin-3-*O*-glucoside equivalents) ANC extract treatment from the 50% blueberry: 50% blackberry wine mixture (ANC50%) significantly expressed the most HNF-1α in Caco-2 cells by increasing its expression by 260% as compared to a media only control. It was also significantly different from the other treatments (P < 0.05) as determined by a Games-Howell post hoc test. The 5 μM ANC extract treatment from the 100% blackberry dealcoholized wine (ANC100%) increased HNF-1α expression by 144%, the 50 μM ANC50% treatment by 142%, and the 50 μM treatment of VitaBlue blueberry ANC extract by 85.2% in Caco-2 cells. These three ANC treatments increased significantly Caco-2 nuclear HNF-1α expression compared to the media only control (P < 0.05), but were not statistically different from each other.

There was a significantly different (P < 0.05) modulation of the relative expression of HNF-1α by pure D3G at 5 μM of 3.19 ± 0.01 and by pure C3G at 5 μM of 1.06 ± 0.03 compared to the control of media-only. From these results (S2 Fig), the contribution to modulate HNF-1α expression by ANC extracts was due to higher abundance of D3G, and less of an effect due to the C3G present.

**Fig 7:**
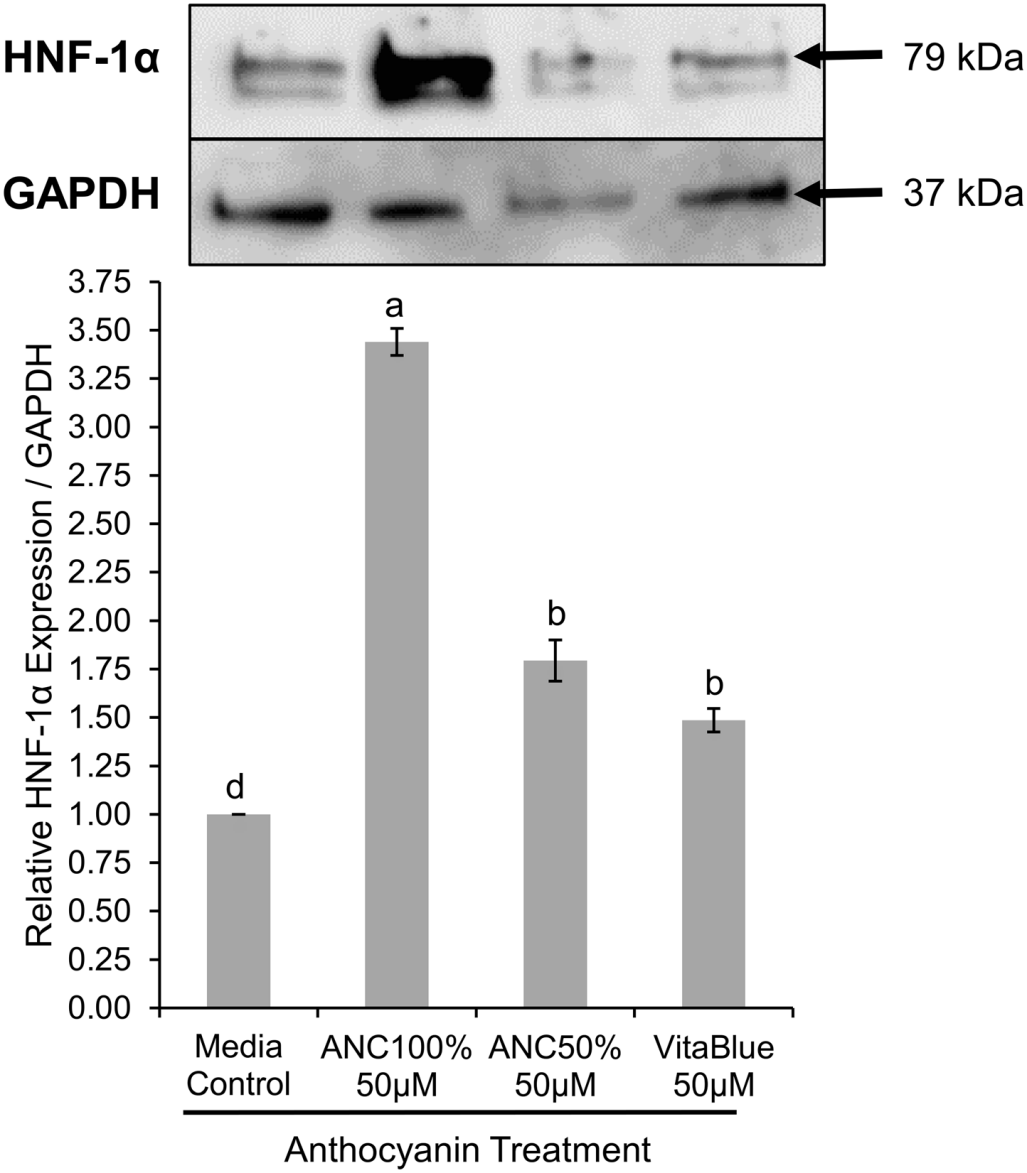
Effects of anthocyanin extracts on nuclear hepatocyte nuclear factor-1α (HNF-1α) expression in normal colon cells. Anthocyanin extract treatments from blueberry and blackberry fermented beverages are named as ANC with the percentage of the beverage mixture that was derived from blackberries (ANC50% = 50% blueberry: 50% blackberry, ANC100% = 100% blackberry). VitaBlue is a commercial blueberry 20% anthocyanin extract. Dosage (μM) is given in cyanidin-3-O-glucoside equivalents, and the media control was from cells grown only in media. Relative expression of nuclear HNF-1α over GAPDH is graphed with the mean ± SEM with different letters indicating significantly different expression values (n ≥ 3, P < 0.02). Western blot bands for HNF-1α (79 kDa) and GAPDH (37 kDa) are displayed directly above the respective column of each treatment or control.

HNF-1α expression was also up-regulated in the nucleus of normal colon cells by all the ANC treatments studied (Fig 7). The 50 μM ANC 100% treatment significantly increased (P < 0.02) HNF-1α expression the most by 243% compared to the media only control in normal colon cells. This increase in HNF-1α expression was also significantly different from the other treatments (P < 0.02) as determined by a Games-Howell post hoc test. The 50 μM ANC 50% (79.4% increase) and 50 μM VitaBlue ANC extract (48.6% increase) treatments also significantly increased (P < 0.02) in healthy colon cell HNF-1α expression compared to the media control.

## Discussion

Computational molecular docking of various berry phenolic compounds and citrus flavonoids to the dimerization domain and transcription domain of HNF-1α showed that anthocyanins and anthocyanidins, commonly found in berries, had on average more interactions and a higher affinity than resveratrol and the citrus flavonoids studied. Delphinidin-3-*O*-arabinoside had the highest affinity for the transcription domain of HNF-1α, indicating that it is most likely to interact with this domain of HNF-1α as compared to the other compounds tested. The best conformation of delphinidin-3-*O*-arabinoside binds to the opposite face of the transcription domain than where the domain binds to DNA, indicating that delphinidin-3-*O*-arabinoside is predicted to interact with this domain even if the domain is interacting with DNA. Anthocyanins with the sugar arabinoside at position 3 might have a higher affinity for the transcription domain of HNF-1α given that malvidin-3-*O*-arabinoside had the second highest affinity for this domain and had nineteen amino acids interactions in common with delphinidin-3-*O*-arabinoside. Anthocyanidins delphinidin and cyanidin had high binding affinities for the transcription domain of HNF-1α, so glycosylation of anthocyanidins to ANC is predicted to not be necessary to interact with this domain.

Analysis of the crystal structure (PDB: 1FG3) of a homodimer of the dimerization domain of HNF-1α interacting with a dimer of the dimerization coactivator DCoH indicates that the amino acids SER6, GLN7, LEU8, GLU11, ALA15, GLU18, and SER19 of the dimerization domain of HNF-1α interact with DCoH. Of those interacting amino acids from the dimerization domain of HNF-1α, GLN7, LEU8, GLU11, and GLU18 are said to be the most important in the interaction between HNF-1α and DCoH [3]. DCoH stabilizes the dimerization of HNF-1α, and it increases HNF-1α’s activity [26]. All the compounds tested in this study are predicted to interact with SER6 out of all the amino acids in the dimerization domain of HNF-1α that interact with DCoH (Table 2). Since SER6 is not as critical for the interaction between HNF-1α and DCoH, it is not predicted that the compounds studied will interfere between the formation of the HNF-1α and DCoH complex.

Delphinidin-3-*O*-aribinoside also had the highest binding affinity for a homodimer of the dimerization domain of HNF-1α. The dimerization domain of HNF-1α exists as a homodimer in functional HNF-1α in cells, and the assembly of the dimerization domain as a homodimer is important for HNF-1α binding to its dimerization coactivator [2,3]. Anthocyanins and anthocyanidins had the highest affinities on average for the homodimer of the dimerization domain indicating that these compounds are likely to interact with both the dimerization and transcription domains in fully functional HNF-1α. Interestingly, flavone had the highest affinity for a single unit of the dimerization domain of HNF-1α. Resveratrol has been previously reported to have a close interaction with a single unit of a mutated dimerization domain of HNF-1α compared to other compounds such as aspirin and retinol [14]. The differences observed between resveratrol and flavone having the best interactions with a single unit of the dimerization domain of HNF-1α in these two studies, might be due to the fact that the wild-type form of the dimerization domain was used in this study instead of a mutated version. This study is the first to study the interactions of flavonoids with HNF-1α. Flavone had only van der Waals interactions with the dimerization domain of HNF-1α indicating that electrostatic interactions, such as hydrogen bonds, are not necessary for compounds to have a high affinity for the dimerization domain of HNF-1α. Citrus flavonoids had high binding affinities for the dimerization domain of HNF-1α compared to the berry compounds studied, but binding affinities of all the compounds tested to the dimerization domain of HNF-1α were relatively low compared to the other structures of HNF-1α analyzed. Most of the compounds studied had a wide variety of interactions and higher binding affinities to the transcription domain of HNF-1α compared to the dimerization domain, making the transcription domain a better target for future interaction studies of berry and citrus compounds with HNF-1α.

The large search space parameters used with this computational methodology showed to provide good predictions of the interactions compounds would have to the HNF-1α structures studied. The experimental and crystallographic conformations of myristic acid had a root-mean square deviation (RMSD) value less than two angstroms (1.458 Å). An RMSD value of less than two angstrom between a known conformation and a conformation acquired through an experimental docking scheme is commonly used in computational molecular docking to determine if a docking scheme can give valid predictions [21]. The differences in the experimental and crystallographic conformations of myristic acid are probably due to fact that the myristic acid ligand used to validate the computational scheme was allowed to rotate as shown by the rotated experimental conformation in Fig 5B. Rotation of ligand bonds allows for a more realistic simulation as compounds are rarely rigid structures in nature.

Nuclear HNF-1α expression of Caco-2 and normal colon cells treated with blackberry and blueberry anthocyanin (ANC) extracts increased significantly for all ANC extract treatments. Increased expression of HNF-1α might have positive effects on cells given that HNF-1α promotes expression of a variety of essential proteins such as glucose-6-phosphatase and albumin [27] and has been regarded as essential for hepatocyte differentiation and maintenance of hepatocyte phenotype [28]. More interestingly, a low dosage of 5 μM (cyanidin-3-*O*-glucoside equivalents) of a 50% blueberry: 50% blackberry dealcoholized wine ANC mixture (ANC50%), increased the expression of HNF-1α significantly more (260%) than a larger dosage of 50 μM and the other treatments studied in Caco-2 cells. Interactions between ANCs in this extract at a high concentration of 50 μM might introduce interactions that decrease expression of HNF-1α. The 100% blackberry ANC (ANC100%) extract is predominantly composed of delphinidin-3-*O*-arabinoside [19], so no interactions between different ANCs are predicted to occur for this treatment like they do for the ANC50% extract. When analyzing the effect of the ANC treatments on HNF-1α expression in normal colon cells, the 50 μM ANC100% extract increased HNF-1α in a way comparable to the 5 μM ANC50% extract in Caco-2 cells. Since delphinidin-3-*O*-arabinoside is present significantly in both extracts [19], it is predicted that delphinidin-3-*O*-arabinoside may be the key compound in increasing HNF-1α nuclear expression. It was confirmed that pure delphinidin-3-*O*-glucoside modulated the relative expression of HNF-1α in more than 3-fold relative to the control in Caco-2 cells. Further evaluation of the interaction effect of pure compounds on the modulation of HNF-1α expression in other cell lines would be a valuable addition to the effects seen through computational modeling.

Given the difference in HNF-1α nuclear expression between the 5 and 50 μM ANC50% treatments in Caco-2 cells, accumulation of ANC other than delphinidin-3-*O*-arabinoside in cells is predicted to decrease HNF-1α expression. Since the 50 μM ANC100% treatment had the greatest increase in HNF-1α expression in normal colon cells, these cells are predicted to have absorbed large amounts of delphinidin-3-*O*-arabinoside compared to ANC that can decrease HNF-1α expression. This also applies to the increase in HNF-1α from the 5 μM ANC100% treatment in Caco-2 cells as it was not statistically different from that of the 50 μM ANC50% treatment (Fig 6). The greater increase of HNF-1α by the 5 μM ANC50% treatment in Caco-2 cells in comparison to the 50 μM ANC100% treatment in normal colon cells is thought be due to the differences in concentration of delphinidin-3-*O*-arabinoside available to the cells and the general capacity of the cells to uptake ANC.

The 20% VitaBlue ANC extract has various organic compounds, so it is difficult to correlate HNF-1α expression to the ANCs in this extract. That being said, the treatment of cells with the VitaBlue ANC extract indicates that the presence of at least 20% blueberry ANCs in an extract may increase nuclear HNF-1α expression. While an increase in nuclear expression of HNF-1α was found, when HNF-1α is over-expressed to non-physiological conditions, its activity is only increased 5-fold with the presence of DCoH [29]. This means that over-expression of HNF-1α does not necessarily mean that there will be a significant increase in its activity.

In conclusion, ANCs and anthocyanidins from berries had high binding affinities for the transcription domain and homodimer of the dimerization domain of HNF-1α, while citrus flavonoids had higher affinities for the dimerization domain of HNF-1α. ANC extracts from blueberries and blackberries significantly increased nuclear HNF-1α expression in Caco-2 cells, with delphinidin-3-*O*-arabinoside being predicted as the responsible compound for the increase of nuclear HNF-1α expression. The wide variety of interactions berry and citrus phenolic compounds have with the known domains of HNF-1α predict that they might regulate nuclear expression of HNF-1α directly through interactions with its domains, but the mechanisms of regulation are not yet fully understood. This is the first study to characterize the interactions of resveratrol and flavonoids with wild-type HNF-1α, and it is the first to report an increased expression of HNF-1α in cells exposed to anthocyanins. Our laboratory will continue studying the activity of HNF-1α in cells exposed to ANCs to find out if the observed increase in nuclear HNF-1α expression by these compounds also increases the transcription of proteins such as DPP-IV and GLUT1 and GLUT2 transporters. This would allow for the observed interactions of this study to be connected to possible pre-transcriptional regulation of proteins associated with type II diabetes.

## Supporting Information

S1 Fig Western blotting membrane images for hepatocyte nuclear factor-1α (HNF-1α) and glyceraldehyde 3-phosphate dehydrogenase (GAPDH). The lanes in each membrane are labeled by the anthocyanin treatment the cells received. Bands that were cropped out in Fig 6 are labeled as “Not Analyzed” for clarity. Anthocyanin extract treatments from blueberry and blackberry fermented beverages are named as ANC with the percentage of the beverage mixture that was derived from blackberries (ANC50% = 50% blueberry: 50% blackberry, ANC100% = 100% blackberry). Dosage (μM) is given in cyanidin-3-O-glucoside equivalents. Thin bands not defined as HNF-1α (79 kDa) or GAPDH (37 kDa) are thought to be from non-specific binding of the antibodies used.
(TIFF)

S2 Fig Effects of pure cyandin-3-*O*-glucoside (C3G) and delphinidin-3-*O*-glucoside (D3G) on nuclear hepatocyte nuclear factor-1α (HNF-1α) expression in Caco-2 cells. Relative expression of nuclear HNF-1α over GAPDH is presented with the mean ± SEM with different letters indicating significantly different expression values (n ≥ 3, P < 0.05). Western blot bands for HNF-1α (79 kDa) and GAPDH (37 kDa) are displayed directly above the respective column of each treatment.
(TIFF)

## References

[1] Chi YI, Frantz JD, Oh BC, Hansen L, Dhe-Paganon S, Shoelson SE. Diabetes mutations delineate an atypical POU domain in HNF-1α. Mol Cell. 2002; 10: 1129–1137. 12453420 10.1016/s1097-2765(02)00704-9

[2] Rose RB, Endrizzi JA, Cronk JD, Holton J, Alber T. High-resolution structure of the HNF-1α dimerization domain. Biochemistry. 2000; 39: 15062–15070. 11106484 10.1021/bi001996t

[3] Rose RB, Bayle JH, Endrizzi JA, Cronk JD, Crabtree GR, Alber T. Structural basis of dimerization, coactivator recognition and MODY3 mutations in HNF-1α. Nat Struct Biol. 2000; 9: 744–748. 10.1038/78966 10966642

[4] Galán M, García-Herrero CM, Azriel S, Gargallo M, Durán M, Gorgojo JJ, et al Differential effects of HNF-1α mutations associated with familial young-onset diabetes on target gene regulation. Mol Med. 2011; 17: 256–265. 10.2119/molmed.2010.00097 21170474 PMC3060974

[5] Gu N, Adachi T, Matsunaga T, Takeda J, Tsujimoto G, et al Mutant HNF-1α and mutant HNF-1β identified in MODY3 and MODY5 downregulate DPP-IV gene expression in Caco-2 cells. Biochem Biophys Res Commun. 2006; 346: 1016–1023. 16781669 10.1016/j.bbrc.2006.06.010

[6] Luni C, Marth JD, Doyle FJ II. Computational modeling of glucose transport in pancreatic β-cells identifies metabolic thresholds and therapeutic targets in diabetes. PLoS ONE. 2012; 7: e53130 10.1371/journal.pone.0053130 23300881 PMC3531366

[7] Pedersen KB, Chhabra KH, Nguyen VK, Xia H, Lazartigues E. The transcription factor HNF1α induces expression of angiotensin-converting enzyme 2 (ACE2) in pancreatic islets from evolutionarily conserved promoter motifs. Biochim Biophys Acta. 2013; 1829: 1225–1235. 10.1016/j.bbagrm.2013.09.007 24100303 PMC3838857

[8] Shih DQ, Bussen M, Sehayek E, Ananthanarayanan M, Shneider BL, Suchy FJ, et al Hepatocyte nuclear factor-1α is an essential regulator of bile acid and plasma cholesterol metabolism. Nature. 2001; 27: 375–382. 10.1038/86871 11279518

[9] O’Brien VP, Bokelmann K, Ramirez J, Jobst K, Ratain MJ, Brockmoller J, Tzvetkov MV. Hepatocyte nuclear factor 1 regulates the expression of the organic cation transporter 1 via binding to an evolutionary conserved region in intron 1 of the OCT1 gene. J Pharmacol Exp Ther. 2013; 347: 181–192. 10.1124/jpet.113.206359 23922447 PMC3781413

[10] Armendariz AD, Krauss RM. Hepatic nuclear factor 1-α: Inflammation, genetics, and atherosclerosis. Curr Opin Lipidol. 2009; 20: 106–111. 10.1097/MOL.0b013e3283295ee9 19280766

[11] Gu N, Tsuda M, Matsunaga T, Adachi T, Yasuda K, Ishihara A, et al Glucose regulation of dipeptidyl peptidase IV gene expression is mediated by hepatocyte nuclear factor-1α in epithelial intestinal cells. Clin Exp Pharmacol Physiol. 2008; 35: 1433–1439. 10.1111/j.1440-1681.2008.05015.x 18671716

[12] Centers for Disease Control and Prevention. National diabetes statistics report: Estimates of diabetes and its burden in the United States. Atlanta, GA: U.S. Department of Health and Human Services; 2014.

[13] Jadav P, Bahekar R, Shah SR, Patel D, Joharapurkar A, Kshirsagar S, et al Long-acting peptidomimetics based DPP-IV inhibitors. Bioorg Med Chem Lett. 2012; 22: 3516–3521. 10.1016/j.bmcl.2012.03.078 22503246

[14] Sridhar GR, Nageswara PV, Kaladhar DS, Devi TU, Kumar SV. In silico docking of HNF-1a receptor ligands. Adv Bioinformatics. 2012; 705435 10.1155/2012/705435 23316227 PMC3535823

[15] Fan J, Johnson MH, Lila MA, Yousef G, de Mejia EG. Berry and citrus phenolic compounds inhibit dipeptidyl peptidase IV: Implications in diabetes management. Evid Based Complement Alternat Med. 2013: 479505 10.1155/2013/479505 24069048 PMC3773436

[16] Johnson JL, Rupasinghe SG, Stefani F, Schuler MA, de Mejia EG. Citrus flavonoids luteolin, apigenin, and quercetin inhibit glycogen synthase kinase-3β enzymatic activity by lowering the interaction energy within the binding cavity. J Med Food. 2011; 14: 325–333. 10.1089/jmf.2010.0310 PMC3123937 21443429

[17] Parmar HS, Jain P, Chauhan DS, Bhinchar MK, Munjal V, Yusuf M, et al DPP-IV inhibitory potential of naringin: An in silico, *in vitro* and *in vivo* study. Diabetes Res Clin Pract. 2012; 97: 105–111. 10.1016/j.diabres.2012.02.011 22410395

[18] Alzaid F, Cheung H-M, Preedy VR, Sharp PA. Regulation of glucose transporter expression in human intestinal Caco-2 cells following exposure to an anthocyanin-rich berry extract. PLoS ONE. 2013; 8: e78932 10.1371/journal.pone.0078932 24236070 PMC3827299

[19] Johnson MH, de Mejia EG, Fan J, Lila MA, Yousef GG. Anthocyanins and proanthocyanidins from blueberry-blackberry fermented beverages inhibit markers of inflammation in macrophages and carbohydrate-utilizing enzymes in vitro. Mol Nutr Food Res. 2013; 57: 1182–1197. 10.1002/mnfr.201200678 23526625

[20] Morris GM, Huey R, Lindstrom W, Sanner MF, Belew RK, Goodsell DS, et al AutoDock4 and AutoDockTools4: Automated docking with selective receptor flexibility. J Comput Chem. 2009; 30: 2785–2791. 10.1002/jcc.21256 19399780 PMC2760638

[21] Trott O, Olson AJ. Autodock Vina: Improving the speed and accuracy of docking with a new scoring function, efficient optimization, and multithreading. J Comput Chem. 2010; 31: 455–461. 10.1002/jcc.21334 19499576 PMC3041641

[22] Bower AM, Real Hernandez LM, Berhow MA, de Mejia EG. Bioactive compounds from culinary herbs inhibit a molecular target for type 2 diabetes management, dipeptidyl peptidase IV. J Agric Food Chem. 2014; 62: 6147–6158. 10.1021/jf500639f 24881464

[23] Pettersen EF, Goddard TD, Huang CC, Couch GS, Greenblatt DM, Meng EC, et al UCSF Chimera—a visualization system for exploratory research and analysis. J Comput Chem. 2004; 25: 1605–1612. 15264254 10.1002/jcc.20084

[24] R Core Team. R: A language and environment for statistical computing. R Foundation for Statistical Computing, Vienna, Austria; 2014 URL http://www.R-project.org/.

[25] Leys C, Ley C, Klein C, Bernard P, Licata L. Detecting outliers: Do not use standard deviation around the mean, use absolute deviation around the median. J Exp Soc Psychol. 2013; 49: 764–766. 10.1016/j.jesp.2013.03.013

[26] Mendel DB, Khavari PA, Conley PB, Graves MK, Hansen LP, Admon A, et al Characterization of a cofactor that regulates dimerization of a mammalian homeodomain protein. Science. 1991; 254: 1762–1767. 1763325 10.1126/science.1763325

[27] Yang YM, Noh K, Han CY, Kim SG. Transactivation of genes encoding for phase II enzymes and phase III transporters by phytochemical antioxidants. Molecules. 2010; 15: 6332–6348. 10.3390/molecules15096332 20877225 PMC6257698

[28] Pelletier L, Rebouissou S, Vignjevic D, Bioulac-Sage P, Zucman-Rossi J. HNF1α inhibition triggers epithelial-mesenchymal transition in human liver cancer cell lines. BMC cancer. 2011; 11: 427 10.1186/1471-2407-11-427 21975049 PMC3203860

[29] Hansen LP, Crabtree GR. Regulation of the HNF-1 homeodomain proteins by DCoH. Curr Opin Genet Dev. 1993; 3: 246–53. 8504250 10.1016/0959-437x(93)90030-s

