# Supplementary figures and images for: Berry Phenolic Compounds Increase Expression of Hepatocyte Nuclear Factor-1α (HNF-1α) in Caco-2 and Normal Colon Cells Due to High Affinities with Transcription and Dimerization Domains of HNF-1α
Source: PLoS One. 2015 Sep 28;10(9):e0138768. doi: 10.1371/journal.pone.0138768 (PMC4587667; doi:10.1371/journal.pone.0138768)

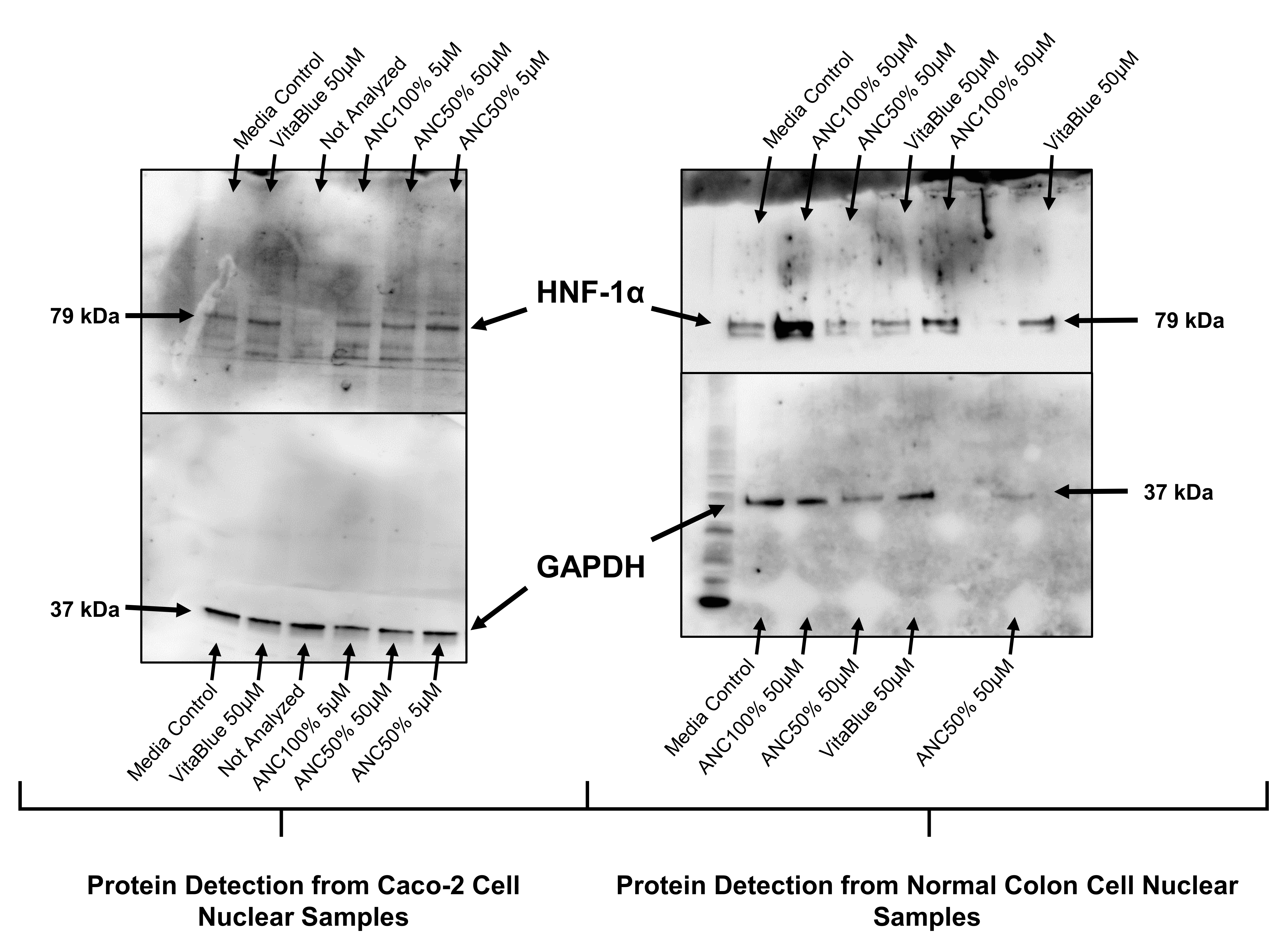

Supplement: S1 Fig — The lanes in each membrane are labeled by the anthocyanin treatment the cells received. Bands that were cropped out in Fig 6 are labeled as “Not Analyzed” for clarity. Anthocyanin extract treatments from blueberry and blackberry fermented beverages are named as ANC with the percentage of the beverage mixture that was derived from blackberries (ANC50% = 50% blueberry: 50% blackberry, ANC100% = 100% blackberry). Dosage (μM) is given in cyanidin-3-O-glucoside equivalents. Thin bands not defined as HNF-1α (79 kDa) or GAPDH (37 kDa) are thought to be from non-specific binding of the antibodies used. (TIFF) [file pone.0138768.s001.tiff]

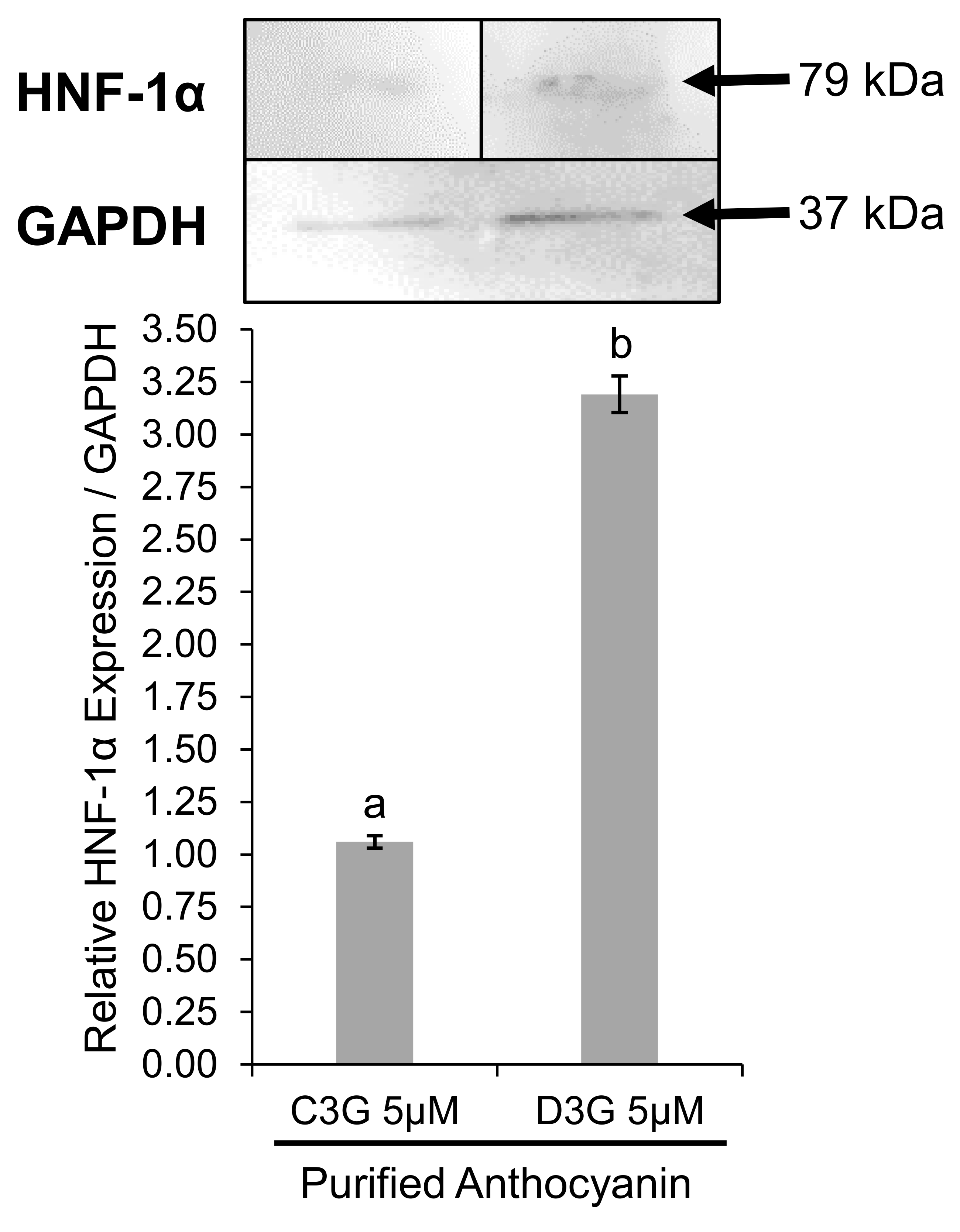

Supplement: S2 Fig — Relative expression of nuclear HNF-1α over GAPDH is presented with the mean ± SEM with different letters indicating significantly different expression values (n ≥ 3, P < 0.05). Western blot bands for HNF-1α (79 kDa) and GAPDH (37 kDa) are displayed directly above the respective column of each treatment. (TIFF) [file pone.0138768.s002.tiff]
